# An App to Support Fathers’ Mental Health and Well-Being: User-Centered Development Study

**DOI:** 10.2196/47968

**Published:** 2023-08-14

**Authors:** Shaun Liverpool, Mia Eisenstadt, Aoife Mulligan Smith, Sofia Kozhevnikova

**Affiliations:** 1 Faculty of Health, Social Care and Medicine Edge Hill University Ormskirk United Kingdom; 2 Evidence Based Practice Unit, Anna Freud National Centre for Children and Families University College London London United Kingdom; 3 fatherli Ltd 103c Camley St, Kings Cross N1C 4PF London United Kingdom; 4 Division of Psychology and Language Sciences Faculty of Brain Sciences University College London London United Kingdom; 5 School of Biosciences University of Nottingham Nottingham United Kingdom

**Keywords:** fathers, parenting, apps, mental health, well-being

## Abstract

**Background:**

Numerous studies describe the popularity and usefulness of parenting programs. In particular, parenting programs are generally viewed as effective for supporting parents’ mental well-being during key transition periods. However, the evidence base for fathers is limited owing to their lack of involvement in parenting programs and scarcity of tailored support.

**Objective:**

This paper aimed to describe the co-design process for a universal digital intervention for fathers (*fatherli*) and the outline of a logic model with its expected outcomes.

**Methods:**

Following established guidelines for co-designing and developing complex interventions, we conducted a nonsystematic review of the available literature to gather key information, developed market surveys to assess fathers’ needs and interests, consulted with key stakeholders to obtain expert opinions, and engaged in a rapid iterative prototyping process with app developers. Each step was summarized, and the information was collated and integrated to inform a logic model and the features of the resulting intervention.

**Results:**

The steps in the co-design process confirmed a need for and interest in a digital intervention for fathers. In response to this finding, *fatherli* was developed, consisting of 5 key features: a discussion forum for anyone to post information about various topics (the forum), a socializing platform for fathers to create and engage with others in small groups about topics or points of shared interest (dad hub), a tool for fathers to find other fathers with shared interests or within the same geographic location (dad finder), a resource for fathers to access up-to-date information about topics that interest them (dad wiki), and a portal to book sessions with coaches who specialize in different topics (dad coaching space). The evidence-based logic model proposes that if *fatherli* is successfully implemented, important outcomes such as increased parental efficacy and mental health help-seeking behaviors may be observed.

**Conclusions:**

We documented the co-design and development process of *fatherli*, which confirmed that it is possible to use input from end users and experts, integrated with theory and research evidence, to create suitable digital well-being interventions for fathers. In general, the key findings suggest that an app that facilitates connection, communication, and psychoeducation may appeal to fathers. Further studies will now focus on acceptability, feasibility, and effectiveness. Feedback gathered during pilot-testing will inform any further developments in the app to increase its applicability to fathers and its usability.

## Introduction

### Background

The importance of fathers’ (referring to anyone with parenting responsibilities who self-identify as male) involvement in parenting for children’s development has been well documented in research [1,2]. However, fathers are often described as being *overlooked* in parenting programs and support for new parents [1,3]. Many parenting programs also report low engagement of fathers, relative to mothers [1,3]. Furthermore, studies have found that many fathers felt excluded and unsupported by health care professionals during pregnancy and the perinatal period [4,5]. Within the research base, fathers’ mental health and well-being during the transition to fatherhood have been described as being under-studied [6]. Despite the potential positive outcomes of having a child, it can be perceived to be stressful for both mothers and fathers, with effects on the couples’ relationship [7,8]. There is also a lack of studies of the experiences of same-sex parents when one or both parents adopt a father’s role or title [9].

Moreover, in the past decade, there is increasing understanding in policy and research regarding the issues that fathers can experience, including stress, burnout, tiredness, postnatal depression, and difficulties with adjusting to the role of fatherhood [10]. However, there is still a paucity of studies of interventions to address specific mental health issues and fathers’ well-being more broadly. Despite some efforts, the focus has been on teaching fathers the skills for parenting via parenting classes, rather than supporting fathers emotionally [11]. Although previous studies highlight that apps and digital parenting interventions are effective for parents, there is little evidence specifically for fathers [12]. Similarly, there is a lack of commercially available apps designed just for fathers. Taken together, there is an urgent need for both the creation and acceptability testing of digital apps for fathers.

Given the barriers to fathers seeking support or accessing parenting programs, apps and web-based support may be a promising low-cost and discreet medium through which fathers can access parenting and well-being support related to fathering. This support may be temporary; long term; or during particular periods, such as during pregnancy, during relationship breakdown, during conflict with a coparent, or when a child is going through particular issues such as bullying or mental health symptoms [13]. A study by Virani et al [14] revealed that only a few apps for fathers have been evaluated (eg, *mDad*, *Milkman*, and *DadTime*). However, it is still unclear whether fathers would benefit from an app to support them with their well-being and parenting. Thus, there is a need to consult users and stakeholders to understand what fathers’ needs are through birth, childhood, and teenage years; what support fathers are currently accessing; and whether fathers will welcome or benefit from forms of digital support and guidance.

### Aims and Objectives

Owing to the lack of readily available, tailored support for fathers, the primary purpose of this study was to report the development process of a universal digital intervention to support fathers with their parenting and mental well-being. As a secondary objective, we also described a preliminary version of a logic model with its expected outcomes.

## Methods

### Theoretical Approaches

#### Overview

The development process for the proposed intervention was guided by the established frameworks for developing and evaluating complex interventions. First, we adhered to the framework proposed by the Medical Research Council, which highlights the importance of exploring the evidence base for interventions, conducting needs assessments with key stakeholders, and modeling the process and outcomes [13]. We also followed the recommendations for designing digital interventions, which suggest that the first phase of the intervention should comprise a transparent development process and a clear modeling of the complex digital intervention before moving to the acceptability and feasibility pilot-testing phase [15,16].

As part of the app development process, we adopted a co-design approach. An important aspect of co-designing is that future users are collaborating with professional experts, such as researchers, fatherhood-related organizations, and developers [17]. Co-design has many benefits including the fact that the usability and early identification and addressing of user needs can increase the likelihood that the service or intervention will become universally acceptable and accessible [18]. More specifically, we were guided by the co-design framework of Sanders and Stappers [19], which outlines 4 interconnected phases—predesign, generative, evaluative, and postdesign phases. In our development process and stakeholder consultations, we focused on the first 2 phases—predesign phase and generative phase—which involves focusing on users’ past, present, and future experiences and then generating ideas regarding user needs, which can inform the new product. A variety of methods were adopted to obtain useful information and evidence for the key features or components of the intervention. It is anticipated that identifying the key features and components through research will bring about positive associations with the desired outcomes and, therefore, address the identified needs [13].

#### Needs Assessment and Consultation Exercises (December 2021)

First, we performed a nonsystematic scoping review of the available evidence about fathers’ mental health and well-being (in particular, separated fathers or nonresident fathers living away from their children), to identify the nature and size of the problem and to guide the choice of intervention components that could overcome some of the challenges. This scoping exercise identified 9 reviews [20-27] including 1 relevant qualitative systematic review [28]. Regarding policy documents, >20 reports have been published by the Fatherhood Institute, focusing on topics such as fatherhood and postnatal depression, fathers’ involvement during the COVID-19 pandemic, and engaging fathers in perinatal care [29]. Other relevant policy documents focus on fathers’ mental health and father-inclusive practice [30]. These were deemed relevant and subsequently informed our decisions throughout the intervention development process. Some of the challenges of conducting studies of the support needs of fathers were summarized in a paper by Tarrant et al [31]. Information from the different documents was summarized (refer to the *Results* section) and notes were maintained as an audit [32].

#### Market Research Surveys (January 2022 to July 2022)

Then, to understand user needs and preferences, 4 market research surveys with open and closed questions were developed and disseminated to an international sample of fathers using snowballing and purposive sharing techniques [33-35]. The first survey aimed to capture fathers’ needs related to their parenting and well-being. The second survey aimed to capture fathers’ interest in a social network or web-based platform to support fathers. A third survey shared the mock screens of an app and asked fathers which features they would be most likely to use, and the strength of interest was captured using a Likert scale ranging from 1 to 5. On the basis of the results of the third survey, a fourth survey aimed to capture further details about fathers’ interests on a small number of features, with mock screens shared via the Marvel app (Marvel App Developments Limited) [36]. Characteristics of all the respondents are presented in Table 1. Images of the tested screens are available in Multimedia Appendices 1-3. Surveys were designed and distributed through Google Forms.

**Table 1:** Demographic data about the participants of the 4 surveys^a^.

Characteristics			Survey 1 (n=71), n (%)		Survey 2 (n=68), n (%)		Survey 3 (n=31), n (%)		Survey 4 (n=26), n (%)
**Number of children (includes both biological and stepchildren)**									
	1	20 (28)		20 (29)		N/A^b^		N/A	
	2	35 (49)		36 (53)		N/A		N/A	
	3	11 (15)		8 (12)		N/A		N/A	
	≥4	4 (6)		4 (6)		N/A		N/A	
	Other	1 (1)		N/A		N/A		N/A	
**Age range (years)**									
	21-30	5 (7)		0 (0)		N/A		N/A	
	31-40	26 (37)		23 (34)		N/A		N/A	
	41-50	31 (44)		34 (50)		N/A		N/A	
	≥51	9 (13)		10 (15)		N/A		N/A	
	Not specified	0 (0)		1 (1)		N/A		N/A	
**Ethnicity or race**									
	White	56 (79)		N/A		28 (90)		N/A	
	Ethnic minority group	14 (20)		N/A		2 (6)		N/A	
	Not specified	1 (1)		N/A		1 (3)		N/A	
**Living arrangement**									
	Typical or intact	48 (68)		N/A		22 (71)		N/A	
	Atypical or separated	23 (32)		N/A		7 (23)		N/A	
	N/A	0 (0)		0 (0)		3 (10)		N/A	
**Employment status**									
	Full time	53 (75)		N/A		N/A		N/A	
	Part time	9 (13)		N/A		N/A		N/A	
	Self-employed	5 (7)		N/A		N/A		N/A	
	Other	4 (6)		N/A		N/A		N/A	
**Relationship status**									
	Married	N/A		40 (59)		N/A		N/A	
	Long-term relationship	N/A		10 (15)		N/A		N/A	
	Separated or divorced	N/A		14 (21)		N/A		N/A	
	Single	N/A		3 (4)		N/A		N/A	
	Other	N/A		1 (1)		N/A		N/A	

^a^The total number of children (n=70) does not include 1 father who reported having an adopted or foster child. All respondents of the survey were fathers but not all were biological fathers.

^b^N/A: not applicable.

### Focus Groups and Consultations (January 2022 to July 2022)

In tandem with the surveys, ME conducted 3 focus groups and individual consultations (n=37) with a purposive sample of key stakeholders (ie, advisers), which included fathers, mental health practitioners, parenting experts, app developers, parenting program developers, and academics specializing in parenting interventions. Snowball sampling was used so individuals were able to provide introductions and recommendations to other stakeholders, within the consultation itself. Consultations were 45 to 60 minutes long and conducted via videoconference calls, and the first author took notes from the consultations [37]. Consultations with fathers as potential service users were recorded where consent was provided, and then, the interviews were transcribed. Characteristics of the advisers are presented in Table 2. Consultants came from a variety of organizations, which included representatives from organizations and research projects, such as Fathers Network Scotland, South London and Maudsley NHS Foundation Trust, Fatherhood Institute, Anna Freud National Centre for Children and Families, Dadly Does It, Following Young Fathers Further, The Fatherhood Awards, Beyond Equality, DadsHouse, and Dads Matter. These consultations provided knowledge and expertise regarding experiences and perceptions about support for fathers in relation to parenting, mental health, and well-being and ideas about how to address some of the barriers to accessing parenting and well-being support and current gaps within existing services. Consultations with fathers as potential service users were conducted to understand needs and potential pain points, which we define as “problems experienced by users that could be addressed by a product” and experiences with current support and social networks used over the web to assist with parenting and well-being.

**Table 2:** Overview of the experts who participated in the stakeholder consultations (n=37).

Stakeholder type	Participants, n (%)
Researchers and parenting experts	10 (27)
Psychologists	2 (5)
Married fathers	4 (11)
Academics, researchers, and practitioners providing interventions to fathers	3 (8)
Developer of a commercial app for fathers	1 (3)
Individuals running interventions for fathers or new fathers	5 (14)
Individuals running programs for divorced and separated fathers or coparenting programs	2 (5)
Divorced or separated fathers	10 (27)

**Figure 1:**
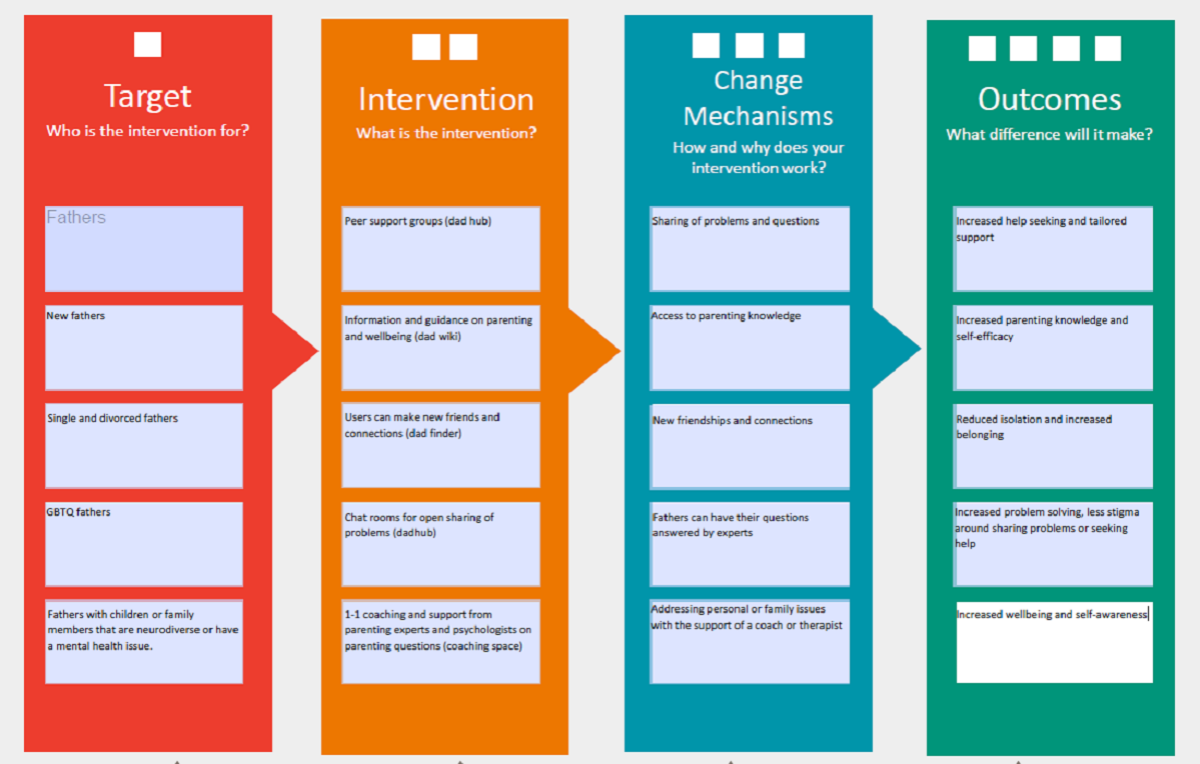
Logic model for fatherli. GBTQ: gay, bisexual, transgender, and queer.

The consultations also provided useful insights to help model the changes expected if the intervention was developed and implemented (refer to Figure 1 in the *Results* section).

### Design and Development Process of the App (August 2022 to November 2022)

A rapid prototyping process was then adopted to go from idea generation to low-fidelity prototype [38]. This was performed through a 4-step iterative process of sharing the prototype with the advisers and potential users to obtain feedback, which was then shared with the app developers to make improvements before sharing the app with the advisers again. Existing literature suggests that samples >15 are necessary for effective user testing [39]. Feedback was presented and discussed during regular meetings to maintain transparency and trustworthiness [40]. Notes were also archived to document an audit trail [41]. A group of 60 fathers was consulted via a messaging platform to review the features and design and then test the beta version of the app before the final app was submitted to app stores. The final version of the intervention is described in the *Results* section and was agreed upon and approved by the advisory group.

### Ethical Considerations, Participation and Anonymity

As recommended by Ahtinen et al [42], ethics committee approval was not sought because stakeholder engagement was deemed to have a low level of risk and the purpose was to determine the usability of the app. Therefore, participants were viewed as collaborators instead of study participants. Stakeholder engagement and user testing did not involve a clinical population, and we did not seek to fully evaluate the intervention at this early stage. According to Ly et al [43], as the stakeholder engagement was not based on a clinical population, there was no reason to register the study within a public trial registry. This was confirmed using the Health Research Authority Decision Making Tool. The outcome of the tool was that this study was not research; evidence of this outcome is provided in Multimedia Appendix 4 (HRA052523) [44]. Nonetheless, all fathers volunteered and consented to be part of the development of the intervention, and any details shared (eg, email addresses) were securely stored and used only for the purpose of informing this study. Anonymity of the fathers was assured by removing information that could lead to identification when producing this paper. The project lead (ME) was prepared to signpost fathers to relevant well-being support and resources, if required. We also viewed similar development studies [43,45-47] in which no formal ethical approvals were required, and we confirmed this with our academic advisers.

### Data Analysis and Synthesis of Information

Findings from the abovementioned steps were summarized, collated, and integrated to inform the logic model and the features of the resulting intervention, as described in the *Results* section. Data were analyzed using Excel (Microsoft Corporation) and R statistical software (R Foundation for Statistical Computing) [48,49]. For quantitative data, the main focus was on descriptive analysis using frequencies and percentages. Chi-square test was used to explore between-group differences where applicable. Qualitative data from the market research surveys were coded deductively using content analysis, and quotes were used to support the overarching themes obtained from the surveys.

Thematic analysis was used to analyze the data from the consultations, looking for themes of meaning or prevalence. To analyze the interviews, the last author used the steps for conducting a reflexive thematic analysis by Braun and Clarke [50]. This involves the researcher reading and familiarizing themselves with the data set by conducting the interviews, checking the quality of the interview notes, and working with the data set to answer previous research questions. The interviews were coded by the last author by giving descriptive labels (codes) to transcript extracts relevant to participants’ experiences of what fathers need, current interventions for fathers, and possible gaps in the existing support for fathers. According to thematic analysis methodology, which focuses on meaning and prevalence in the selection of relevant quotes, quotes were selected based on the relevance that they had to the research question and, in this case, whether they gave indications about what fathers would want or not want from an app or platform (we did not have sufficient qualitative data to analyze them in terms of prevalence).

Content analysis was used to analyze the data obtained from the surveys. According to Hsieh and Shannon [51], qualitative content analysis involves using a systematic coding technique to classify the content of text data and find themes or patterns; this research method allows for the subjective interpretation of that data’s content. The coding categories used in inductive content analysis are directly and inductively derived from the raw data. By letting the categories and category names “flow from the data,” researchers avoid adopting predefined categories [52]. All authors were involved in the analysis of the data. Data analysis aimed to give new insights about fathers’ current experiences of support and social networks (surveys 1 and 2) and what they might think about a new app or platform for fathers (surveys 3 and 4).

To aid with the design of the app, key outputs in the form of summary notes were used to further inform the features and components of the intervention.

## Results

### Summary of the Findings From the Nonsystematic Scoping Review

Although some studies report that parents’ quality of life improves in the year after childbirth [53], studies often highlight that fathers are at risk of a range of mental health issues that include postnatal depression, postnatal anxiety, postnatal psychosis, stress, and burnout [25]. Similarly, fathers who go through divorce and separation are at risk of a range of issues such as addiction and increased risk of mortality [54]. A systematic review by Baldwin et al [25] on mental health and well-being during the transition to fatherhood found that across 132 studies, fathers struggled with new fatherhood identity, competing challenges, negative feelings and fears, stress and coping, and lack of support with fatherhood. The review suggested that role restrictions led to stress and that fathers used denial or escapism such as working long hours to manage. Other reviews also highlighted the negative impact of paternal postnatal depression and being a nonresident father [18,20,44,48].

Regarding the existing interventions for fathers, a review reported that there is a lack of evidence and that more studies are needed to determine the influence of interventions over time and the optimal engagement required [26]. Studies also identified barriers to fathers’ participation in parenting programs, with suggestions that there needs to be active promotion of interventions for fathers with bespoke services [20,21]. To the best of our knowledge, no systematic reviews of digital interventions such as apps for fathers were available at the time of our search.

Both policy documents and research papers suggested that existing initiatives primarily focused on support for mothers and children [55], and many have advocated for father-inclusive practice but with limited success. Barriers to father-inclusive practice included personal, organizational, strategic, and societal factors [56]. A recent report titled “Fathers Reaching Out – Why Dads Matter: 10 years of Findings on the Importance of fathers’ Mental Health in the Perinatal period” found that fathers would benefit from more support for mental health and well-being during key transition periods [48]. The authors recommended that supporting mental and emotional needs of fathers, preparing fathers for the adjustment of fatherhood, and making it easy for fathers to access support is urgently needed [57].

### Findings From Survey 1—Fathers’ Needs and Experiences

Of the 71 participants who completed the first survey, 54 (76%) reported that they needed additional support. Of 54 participants who needed additional support, 43 (80%) were White and 42 (78%) were in full-time employment. See Table 1 for further demographic details of the sample. A chi-square test was conducted to compare fathers who needed additional help with those who reported “no.” No significant difference was found in a desire for additional support between fathers with an atypical family composition and fathers with a typical composition (ie, traditional nuclear family; *χ*^2^_4_=5.8, *P*=.21).

Out of the 71 fathers, 45 (63%) respondents thought that fathers do not receive as much support as mothers in parenting. Of the 71 fathers, 29 (41%) reported difficulty in managing discipline and behavior, 13 (18%) found it difficult to make decisions with their child, and 12 (17%) had difficulty in managing mental health issues. Fathers were also asked where they accessed parenting support resources; of the 71 fathers, 33 (46%) reported that they used websites, 25 (35%) referred to the use of social media, and 22 (31%) reported that they did not have access to support.

When asked if they would like more support, fathers described a range of different needs for support and the importance of acknowledging the diversity in family types:

Peer support groups would be good, local places you can hang out with dads. I’m often one of a very few men in playgroups or clubs.

I would like forms and surveys like this to acknowledge my family more. Instead of having options like mother and father it wouldn’t hurt to put other parent or carer. Families with two dads are often made to feel they have to use the OTHER box in surveys or cross out the mother box on forms

the reverse is true for same sex female couples

Help with getting more time with child. Secondly, support with my parenting decisions/approach being validated by close family and the child’s mother.

From the survey, it was understood that there were many topics that fathers would like more information about and support with regarding parenting and coparenting and that there was a wide range of needs for support that varied with circumstance. It was still unclear the extent that a social network or social app would be useful for fathers; however, some fathers described wanting to connect with other fathers. A second survey was devised to understand fathers’ interest in a possible social network only for fathers.

### Findings From Survey 2—Fathers’ Experiences of Social Networks

A total of 68 fathers responded to the second survey. Of these 68 fathers, 23 (34%) reported that they wanted to connect with other fathers on a father-only social network. However, 71% (48/68) of the participants said that they would like to know more about local activities for fathers and children in the local area. Of the 68 fathers, 58 (85%) said that having children of similar ages was an important factor when connecting with other fathers; 45 (66%) said that being local was important; and 42 (62%) reported that shared interests, hobbies, and values were important factors when connecting with other fathers. Of the 68 respondents, 36 (53%) said that it appealed to them to help other fathers and to share tips. The survey also revealed that, of the 68 fathers, 52 (76%) wanted to see more content related to activities for children and parents; 35 (51%) wanted content related to parental mental health and well-being; and 30 (44%) desired content related to practical parenting support for young children, such as setting boundaries, discipline, bonding, and communication. Regarding the question of paying for a subscription service, of the 68 respondents, 19 (28%) reported that they would pay US $2.49 to US $6.49 per month, and 11 (16%) reported that they would pay US $6.49 to US $12.99 per month to receive additional personalized coaching via the app.

When asked to comment about existing social networks such as Facebook or LinkedIn to connect with others from the perspective as a man or a father, some fathers described not using them or using them solely for work, whereas others felt that that they lacked quality or did not feel inclusive for fathers:

Existing networks for parents can feel exclusionary of fathers and some even have mum in their name.

Where to start?! I think wide open networks are awful. My only good experiences are with highly controlled highly edited forms of social media that emphasise community and progressive values.

From the qualitative data, it was interpreted that some fathers felt excluded from existing programs and platforms and that new platforms needed to be carefully moderated. Overall, from the survey data, it was concluded that there was interest in a social network but that it needed to be sufficiently distinct from existing large networking platforms to be valuable for fathers. It was also concluded that there was a need for high-quality information about parenting, coparenting, relationships, and well-being topics but that how the information could be delivered might take different forms.

### Findings From Consultations With Married, Separated, and Divorced Fathers

Divorced and separated fathers discussed the issue of terminology and how although the term might be accurate, they themselves did not use those terms in an everyday context to describe themselves, instead using terms such as “father” or “dad” or describing their relationship status as “divorced or separated.” Single and divorced fathers discussed the difficult transition period while going through separation and divorce, which included stressors such as finding somewhere to live for them and their child, learning to parent alone, feelings of sadness for a lost long-term partner, relationship, and loss of the family home. Divorced and separated fathers had mixed experiences regarding the extent to which they found the practical side of looking after a child or their children alone (such as cooking and laundry) and mixed experiences regarding the extent to which they were able to coparent with their former partner, with varying levels of conflict and cooperation. Some divorced fathers spoke about feeling excluded from the family home and sadness at the loss of having a bigger role in their children’s lives; they identified Christmas holidays and birthdays as difficult times to navigate regarding access to their child and possible feelings of loneliness. Individuals who identified as a single father described feelings of loneliness and wanting to meet other fathers who were also going through a difficult divorce to make friends and arrange playdates. They also discussed wanting support to find new activities for their children. Married fathers described how their partner would often lead in making childcare arrangements and finding activities and information. Some married fathers wanted more resources available to them that were designed for fathers. Some new fathers described wanting to access support and information locally in their communities and make new friends.

Following the literature review, quantitative research, and qualitative research, the last author designed mock screens that would be an initial prototype of a social app for fathers that would aim to support fathers with their parenting and well-being. These mock screens were shared via a link with a survey using purposive sampling.

### Findings From Survey 3—Initial App Feedback (July 2022)

A total of 25 fathers responded to the third survey to review 6 mock screens for an app prototype for fathers. Respondents were asked about what they wanted to learn about in the app, within the 3 broad categories of “me,” “my child,” and “my relationship.” Of the 25 respondents, 19 (76%) wanted to see content about child cognition. Child mental health and children’s nutrition were the next most popular categories, with 68% (17/25) and 64% (16/25) of respondents expressing interest, respectively. Within the “me” category of the app, of the 25 fathers, 19 (76%) said that they would like content about well-being, 16 (64%) wished to see content about finance and jobs, 12 (48%) wanted to see content about sports, and 9 (36%) wanted to see content about fitness. The main reason that fathers wanted to join the community (14/25, 56%) was to use the chat function within the app, and 20% (5/25) of them said that they would like to use a schedule function to meet other fathers. The remaining 24% (6/25) of the fathers said that they would like to use both features. Some fathers also made recommendations for improvements for a social platform:

A community large enough that there were regular posts, comments, success stories, etc. that related to me. I would check much more often if I felt like there was the chance that I would develop a meaningful friendship as a result of the community.

Make it social, tap into the dada competitive spirit.

[In relation to a potential goal setting feature] My experience is that the concept of setting a goal will/could be much stronger if in the context of a supportive peer group. What examples will be given to help the initial “freeze” / “writer’s block?” E.g., I wonder if everyone understands the concept of a milestone?

From this qualitative data, it was understood that the quality of interaction was important to potential users, that users wanted to develop friendships, that competition could be a way to promote engagement, and that achieving goals could be more effective if done as a group. However, some of these qualitative data would require further studies to understand how widely these views are shared among fathers and potential app users.

The findings from this survey were then analyzed to reduce the number of features in the app based on user preferences. The features that fathers rated that they were most likely to use were selected for the second iteration of the prototype (based on their answers to the question, “What are your three preferred features?”). The implication of this was that the features—goal setting, games, reviewing activities or service providers, and leader board of dad points—were not included in the second version of the prototype. The remaining features were dad hub, dad wiki, feed or for you page, communities, and top 10 reviews.

### Findings From Survey 4—Feedback About a Mock Prototype (August 2022)

In this survey, fathers were shown a smaller set of mock screens based on the previous feedback and including the following features: communities, for you page, forum, dad hub, and top 10 reviews. When asked whether fathers would want to use the content of the app, 71% (22/31) said that they were very likely to use this feature. Most respondents (23/31, 74%) reported that they were likely to join a community or read what other fathers were saying on the communities feature of the app. When asked how likely fathers were to post a question on a forum, of the 31 respondents, 15 (48%) said that they were likely to post, 9 (29%) reported that they would be unlikely to post on the forum, and 7 (23%) described that they were unsure if they would post.

### Implications

From the results of the survey, there was a level of interest and positive feedback from users regarding specific features of the app. The results from the open and closed questions in the survey were reviewed and these features were then used to form a technical specification document for developers to build a beta version of the app on the low-code platform, Bubble [58]. The last author had a series of consultations with the development team to design the app and user experience drawing on the results of the surveys.

### Summary of Feedback From Stakeholder Consultations

Advisers expressed a need for more support for fathers, particularly after the pandemic, with more fathers undertaking flexible working hours to spend more time with their families. Several academics and mental health advocates felt that the issue of fathers’ mental health was overlooked within perinatal and mental health services and confirmed a need for father-inclusive practice. Academics described how fathers were often unlikely to use parenting interventions, but some researchers were finding some success with engaging fathers in coparenting interventions. Some organizations that focused on supporting single fathers did so through the provision of free legal advice and commented that fathers often needed mental health support but lacked access to it. Some single fathers discussed about loneliness; isolation; wanting to connect with other fathers similar to them, in their local areas; and needing additional support when recovering from divorce. Across a range of interviews, stakeholders discussed that fathers may be reluctant to openly seek help owing to factors of stigma and shame and ideas of masculinity that values stoicism and self-reliance. Academics suggested that providing support to men and fathers requires different methods and strategies than those used with women and mothers. At least 4 experts talked about how fathers are often left out of mainstream perinatal services, especially after childbirth, when the focus is on the mother, and the importance of father-inclusive practice when supporting parents. Some psychologists also mentioned an absence of programs that supported fathers to reflect about their own experiences of being parented as preparation for the role of becoming a father.

### Logic Model for the Proposed Intervention

From the literature reviews, 4 surveys, and consultations with stakeholders, the final and first authors (ME and SL) synthesized the input into a logic model (Figure 1) and then used this to develop the app prototype. The resulting logic model outlines the target population (ie, fathers) and demonstrates how the intervention (ie, the *fatherli* app), which consists of peer support groups, information and articles, ways to meet new fathers and form friendships, and access to one-to-one coaching can result in reduced loneliness, more parenting confidence, new parenting skills, and increased help seeking if successfully implemented.

### Outcomes From the Development Process

**Figure 2:**
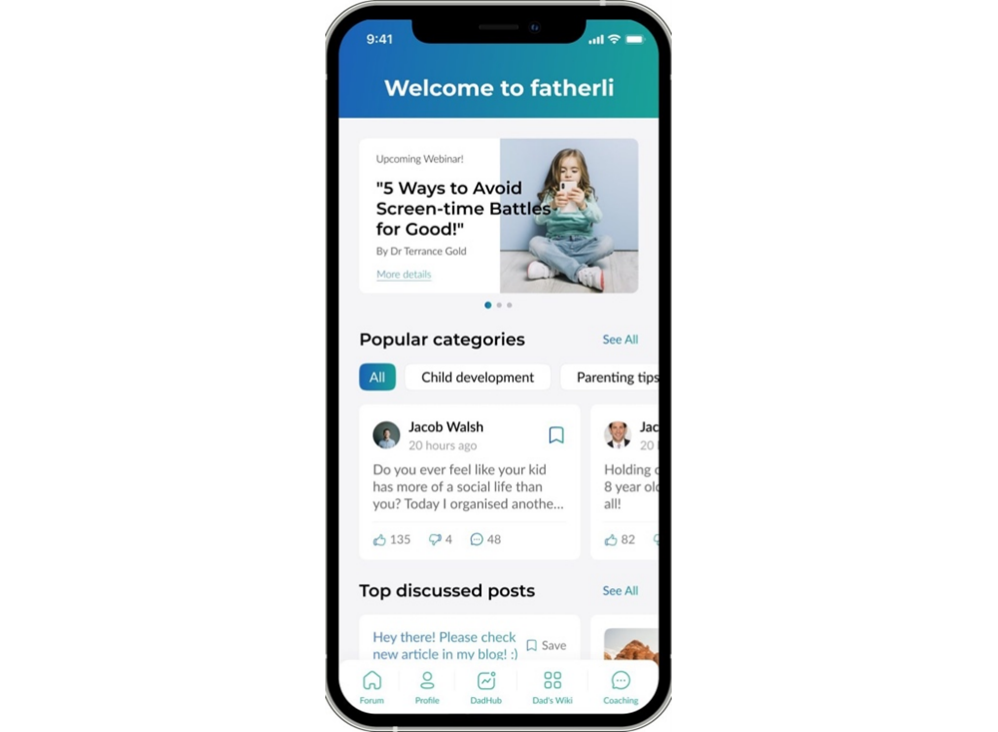
The forum on the fatherli app.

**Figure 3:**
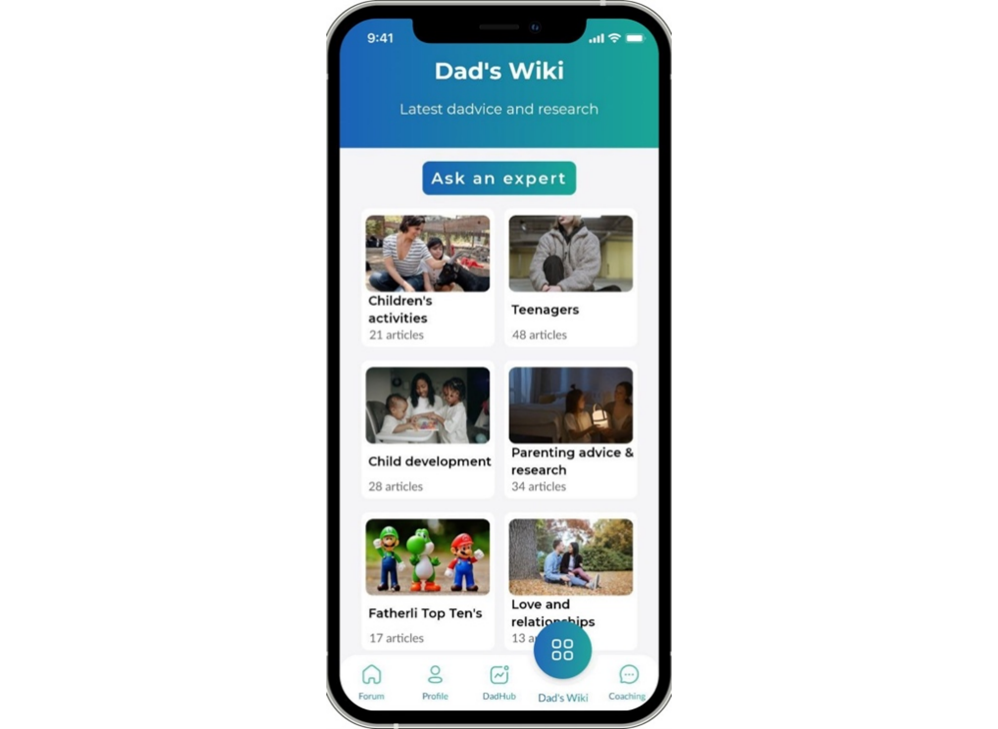
The dad’s wiki feature on the fatherli app.

**Figure 4:**
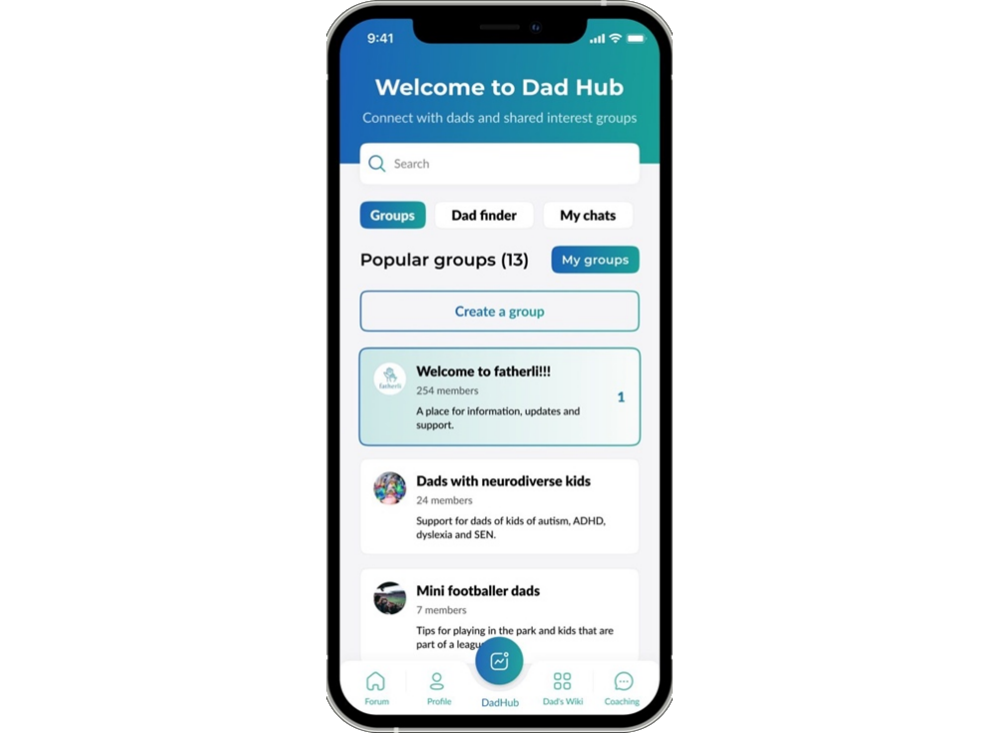
The dad hub on the fatherli app.

**Figure 5:**
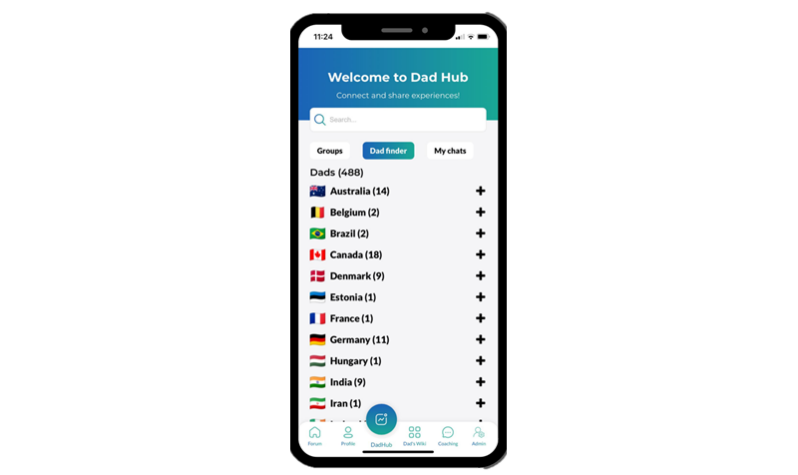
The dad finder feature on the fatherli app.

**Figure 6:**
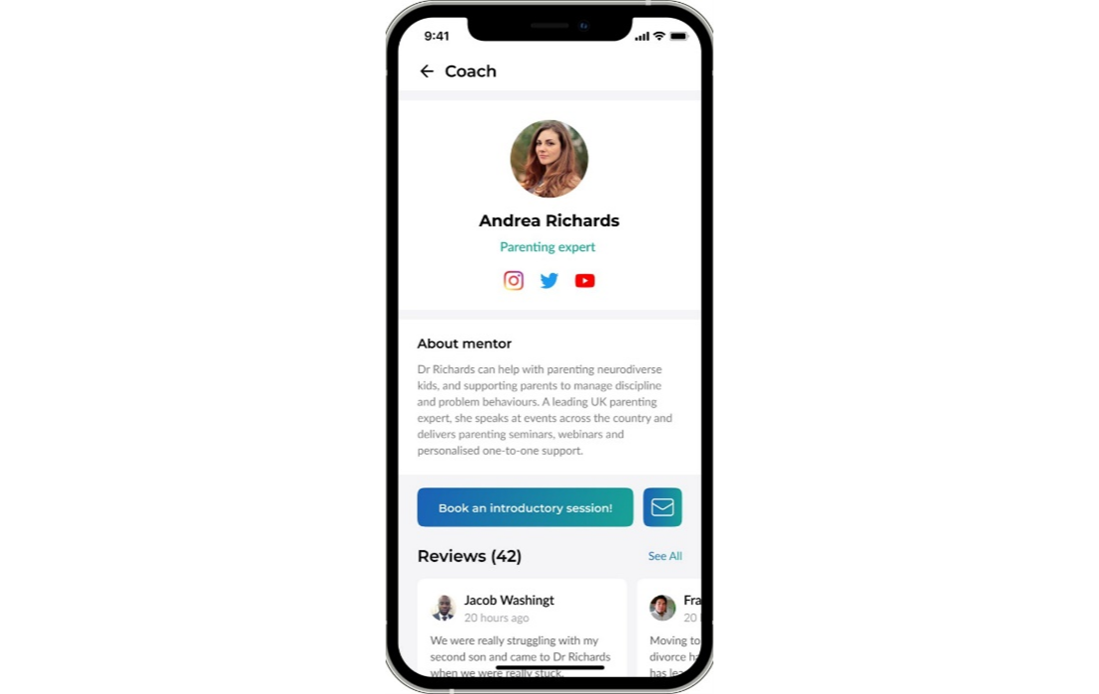
The dad coaching feature on the fatherli app.

The key features of the final app prototype (referred to as *fatherli*) included the forum, dad wiki, ask an expert and a coaching space, dad hub, and dad finder. A descriptive overview of the features is presented in the following sections (Figures 2-6).

#### The Forum

The forum (Figure 2) is an informal place for fathers to post about different topics and comment and reply to others’ posts about a range of topics. The forum can be viewed and sorted into sections (eg, separated or divorced fathers, married fathers, and new fathers), and it also functions as the home screen.

#### Dad Wiki

Dad wiki (Figure 3) includes blog posts and articles about a range of topics written by fathers themselves and by psychologists and postgraduate psychology students. Topics include “top tens” (topics) for fathers and information about baby and child development, parenting research summaries, fathers’ well-being, and love and relationships. All posts represent the views of the writer, and the content is monitored and approved by the app owner (ME) to ensure that misleading opinions that can be harmful are not introduced into the app.

#### Dad Hub

Dad hub (Figure 4) is a place for fathers to join existing groups and create new groups, so that fathers can meet over the web in discussion forums. Groups can be about a topic of shared interest or shared point of commonality, such as parenting neurodiverse children, sports, or being a single parent.

#### Dad Finder

Dad finder (Figure 5) includes a list of users of the app, sorted by geographic location. Therefore, fathers can follow other fathers and look at each other’s interests or posts given on their profiles. By using this feature, fathers could also invite others with similar interest to form new groups or join existing ones.

#### Dad Coaching

Dad coaching (Figure 6) includes a list of coaches and therapists with different areas of expertise. Users can read reviews of the coach’s profiles and then make appointments. By using this feature, fathers could begin a regular coaching relationship with a coach or therapist.

### Feedback About a Working Prototype

Once these features had been created within 1 app on the low-code development platform, Bubble, fathers from the advisory group tested the app on the Bubble website, before it was converted into a native app and submitted to the app stores. Users gave feedback about specific bugs and the need for additional small features such as being able to reply to a comment and edit, delete, or report a comment; adding a moderator to the group; being able to add group descriptions and view group content before joining; improving the functionality of the dad wiki and forum; and making the privacy policy easy to read. The *fatherli* app was released in the app stores in February 2023.

## Discussion

### Principal Findings

This paper documents in detail the development of an evidence-based app and describes the logic model for an intervention to support fathers’ mental health and well-being. The outcomes from the process outlined previously resulted in an app (referred to as *fatherli*) that was co-designed by fathers and informed by experts. The key components of the app are (1) a discussion forum for anyone to post information about various topics (the forum), (2) a socializing platform for fathers to create and engage with others in small groups about topics or points of shared interest (dad hub), (3) a tool for fathers to find other fathers with shared interests or within the same geographic location (dad finder), (4) a resource for fathers to access up-to-date information about topics that interest them (dad wiki), and (5) a portal to book sessions with coaches and therapists who specialize in different topics (dad coaching space).

During the co-design and coproduction process outlined by the co-design framework [19], the need to offer further support for fathers became clear. The review of the available evidence and discussions with experts confirmed that fathers experience a lack of support with parenthood, difficulties in adjusting to the role of parenthood, and a lack of a places to go for support. Therefore, they may experience mental health difficulties such as burnout, depression, and anxiety, which can affect their mental health and well-being [1,6]. The resulting key components of the app generally address the gaps identified in previous reviews of parenting interventions [19-21,45,53]. There were consistent calls for additional support for fathers and a dearth of interventions that addresses mental health among men in a way that is appropriate for them [30]. The needs of fathers also appeared to be unique as suggested by Buckelew et al [59] and Featherstone [60]. This was highlighted in surveys 1 and 2, when fathers indicated that they wanted support with discipline behaviors, children’s mental health, and coparenting, which is not always incorporated in typical parenting interventions [21]. Therefore, the co-design process we adopted allowed us to directly respond to the needs of fathers, as confirmed in survey 1. The second survey also aligned with other studies, which suggests that fathers enjoy opportunities to connect with other fathers [61]. The survey also confirmed that many fathers will welcome information about parenting from experts. However, the fathers in our sample were not keen about an intervention that was orientated toward mothers, which is consistent with the views of Bayley et al [21]. At times, it is possible that fathers put the needs of the child and the family above their own emotional needs and, therefore, may not be ready to admit or engage with their feelings through formal interventions or professional programs based on standardized mental health care models. Therefore, the proposed logic model for *fatherli* could highlight key mechanisms that are specific to fathers’ needs and tailored interventions. Together, the abovementioned findings also strengthen calls to implement technology to support innovative interventions because of its potential to connect people from different geographic locations, enhance accessibility, and reduce stigma [62]. Furthermore, digital interventions also allow users to engage at their own pace [63]. This builds on and strengthens the existing literature that promotes the co-design and coproduction of digital interventions to support mental health and well-being [64,65].

### Strengths and Limitations

An important strength of the development process of *fatherli* is the approach adopted, which was guided by established frameworks for designing complex interventions [16,66]. To the best of our knowledge, this is the first paper to report the development of a complex digital intervention aiming to support fathers through networking and use of popular posts. We also explicitly reported the development process, which experts recommend is an import step when designing interventions [66]. We can confirm that documenting the early stages of the development of an intervention can be useful to facilitate shared knowledge [67]. This is an important contribution to knowledge because we were unable to find any details or references about the development of similar interventions. Another strength of this development process was the ability to include triangulation [68]. We were able to integrate information from previous literature with primary data from fathers (n=4 surveys) and expert discussions (n=37) while following guidelines for an effective co-design process [17,18]. Although the number of fathers completing the surveys varied at each stage, we were consistently above the recommended number of participants for development and user testing of digital technologies [69]. It is possible that although fathers were interested in the intervention itself, they did not always have the capacity to provide feedback [70].

Despite the strengths, some limitations can be acknowledged. First, a rigorous systematic literature review was not conducted to identify the evidence base to support the mental health and well-being needs of fathers. Therefore, some key details could have been missed, and the literature highlighting a need for an intervention to support fathers’ mental health and well-being could have been biased. Nonetheless, the literature we identified consistently highlighted the limited available evidence in the area, which negated a need to conduct a full systematic review and meta-analysis [71]. Another possible limitation is the demographic characteristics of the sample of fathers and experts involved in the co-design process. Although best efforts were made to engage a diverse group of people, our process could have been influenced by a biased sample of individuals who are willing to volunteer their time and expertise to inform research [72]. Similarly, links to the surveys were shared via social media and authors’ networks, making it difficult to estimate the response rates. Therefore, key voices could have been missed during the development process.

### Implications and Recommendations

Interventions addressing the mental health and well-being of fathers could replicate this development process if *fatherli* is found to be effective in later studies. The dearth of evidence-based interventions targeted at fathers and the low engagement with other parenting programs alongside the views of fathers obtained throughout the development process of *fatherli* suggests that fathers would welcome app-based support [73]. In addition, developing an intervention that encourages fathers to connect with other fathers and to engage with materials around effective parenting practices could be an important step in supporting the mental health and well-being of men.

In keeping with the recommendations from the guidelines used to inform the development process [66], *fatherli* will now be tested in a pilot and feasibility phase and then be scaled up to an effectiveness trial to explore the potential outcomes among fathers after using the app. Preliminary discussions with fathers indicated that the intervention itself is generally acceptable [74]. The early testing phases also indicated that there is scope for further development of *fatherli*. Feedback is constantly being integrated into refinements of the intervention and plans for further studies. The preliminary feedback will be considered alongside ongoing advancements in the field [75].

### Conclusions

Adhering to guidelines and recommendation for co-designing and coproducing interventions helped us to develop a complex intervention called *fatherli*. The *fatherli* app aims to support the mental health and well-being of fathers through different activities such as social connections. The development process outlined in this paper describes the multidimensional approach adopted, including exploration of existing literature, theoretical underpinnings, and stakeholder input. The resulting intervention demonstrates and confirms that it is possible to use input from end users and experts, integrated with theory and research evidence, to create suitable digital well-being interventions for fathers. Considering the limitations of this process, further studies will now focus on acceptability, feasibility, and effectiveness. This paper documents the co-design process of *fatherli* and offers preliminary insights into the mental health support needs of fathers such as connection, communication, and psychoeducation. The lessons learned from this process may inform the development of other universal digital interventions to support fathers.

## Appendix

Multimedia Appendix 1 Mock screen in Marvel app, “the dad hub,” shared with users for feedback in survey 3.

Multimedia Appendix 2 Mock screen of the “communities” feature in Marvel app shared with users for feedback in survey 3.

Multimedia Appendix 3 Mock screen in Marvel app, “fatherli games,” shared with users for feedback in survey 3.

Multimedia Appendix 4 Heath Research Authority Decision Tool assessment outcome.

## Data Availability

The data sets generated and analyzed during this study are available from the corresponding author upon reasonable request.
