# Supplementary figures and images for: An App to Support Fathers’ Mental Health and Well-Being: User-Centered Development Study
Source: JMIR Form Res. 2023 Aug 14;7:e47968. doi: 10.2196/47968 (PMC10463090; doi:10.2196/47968)

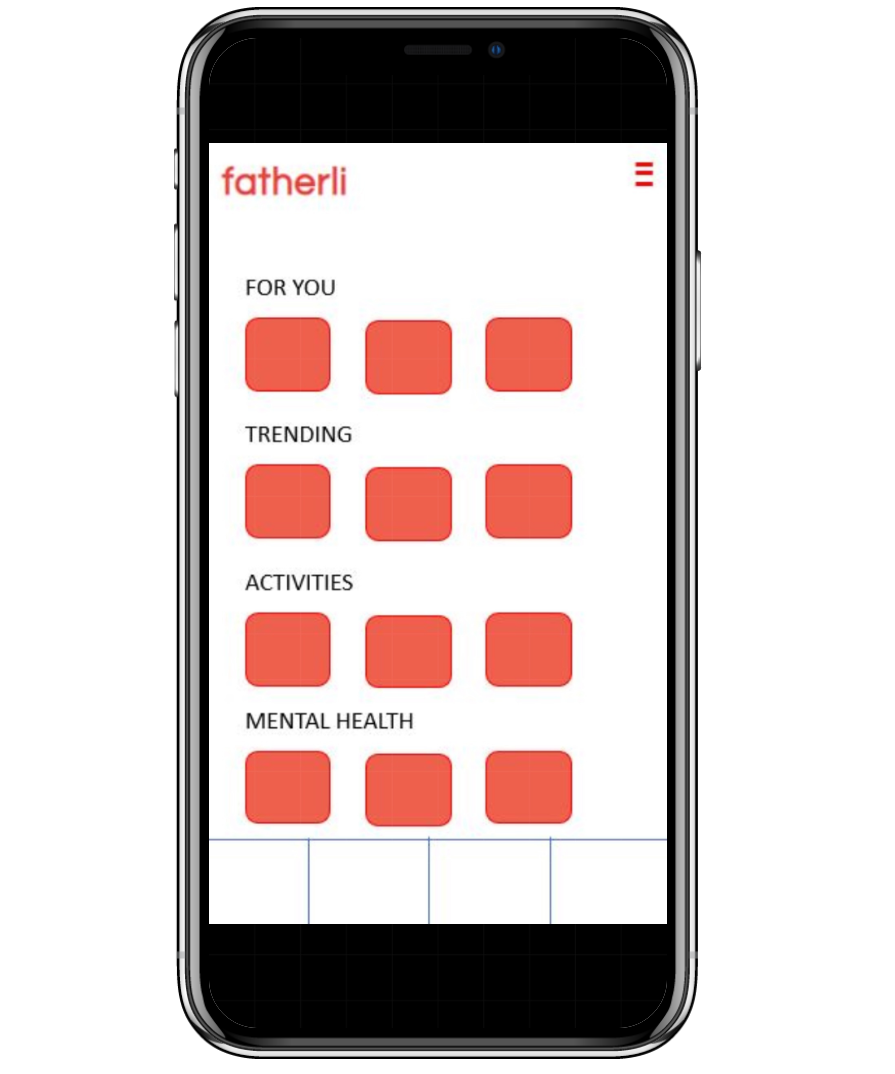

Supplement: Multimedia Appendix 1 [file formative_v7i1e47968_app1.png]

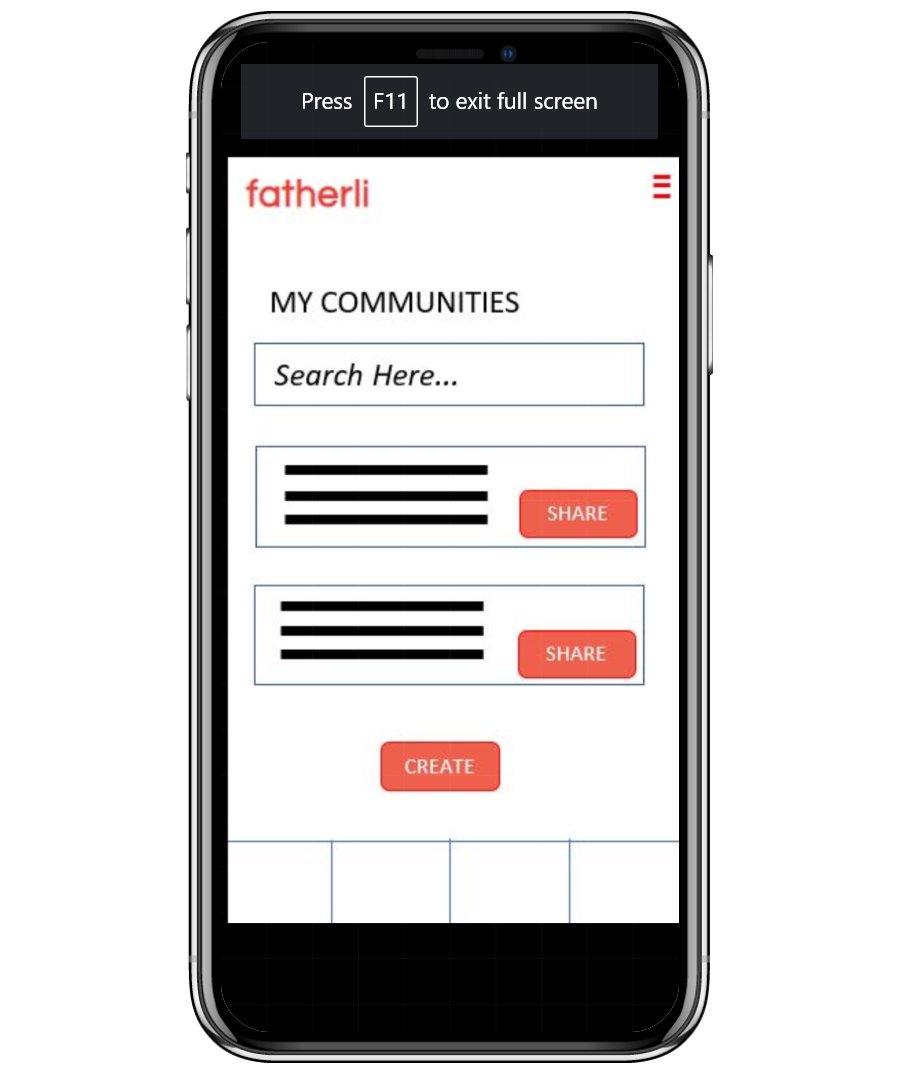

Supplement: Multimedia Appendix 2 [file formative_v7i1e47968_app2.png]

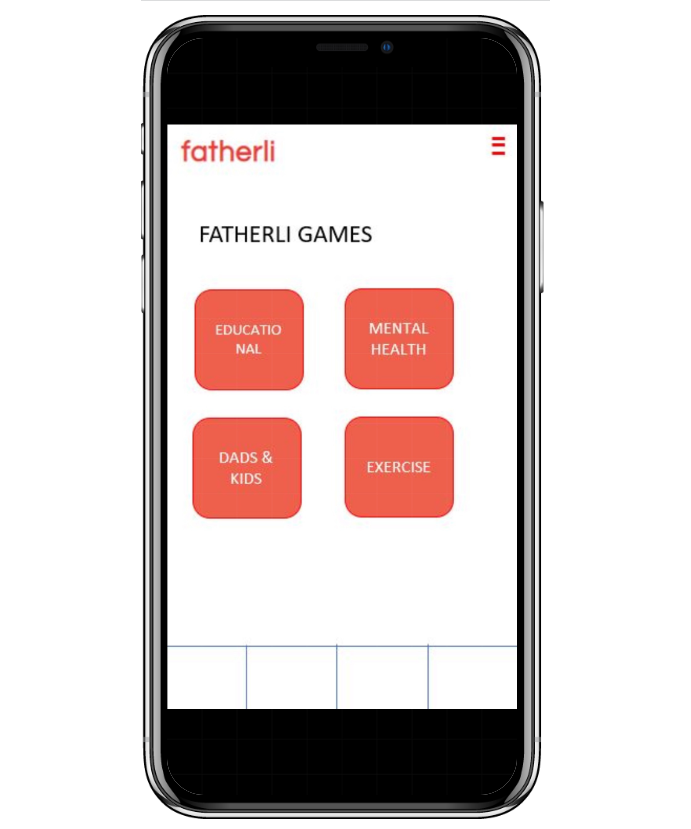

Supplement: Multimedia Appendix 3 [file formative_v7i1e47968_app3.png]
